# Clinical significance of coagulation biomarkers in venous pressure therapy for preventing lower-extremity deep venous thromboembolism: A meta-analysis

**DOI:** 10.5937/jomb0-61632

**Published:** 2026-01-28

**Authors:** Jingrong Niu, Shuang Zhou, Lijin Zhang, Chunmin Li, Hualiang Ren

**Affiliations:** 1 Beijing Chaoyang Hospital, Capital Medical University, Department of Vascular Surgery, Beijing, China

**Keywords:** coagulation biomarkers, fibrinogen, D-dimer, APTT, venous thromboembolism, laboratory diagnostics, biomarkeri koagulacije, fibrinogen, D-dimer, APTT, venska tromboembolija, laboratorijska dijagnostika

## Abstract

**Background:**

To evaluate the biochemical and clinical significance of venous pressure therapy in preventing venous thromboembolism (VTE) through analysis of coagulation and fibrinolysis biomarkers, including fibrinogen (FIB), D-dimer (D-D), and activated partial thromboplastin time (APTT).

**Methods:**

Randomized controlled trials published between 2013 and 2025 were systematically retrieved from PubMed, CNKI, VIP, and Wanfang databases. Eligible studies investigated venous pressure therapy and reported coagulation-related indices. Pooled effect sizes were calculated for key biochemical markers (FIB, D-D, APTT, PT, TT) and venous hemodynamic outcomes.

**Results:**

Nineteen clinical studies met inclusion criteria. Meta-analysis revealed that venous pressure therapy significantly reduced plasma FIB and D-D levels, prolonged APTT, prothrombin time (PT), and thrombin time (TT), and improved venous blood flow velocity. These changes reflect improved anticoagulant activity, enhanced fibrinolysis, and reduced risk of thrombosis. Importantly, the observed modulation of biochemical markers correlated with a lower incidence of lower-extremity deep venous thrombosis.

**Conclusions:**

Venous pressure therapy favorably alters coagulation and fibrinolytic biomarkers, underscoring their diagnostic value in monitoring therapeutic efficacy and thrombotic risk. These findings highlight the critical role of laboratory indices in guiding the prevention and management of VTE, supporting their integration into standardized clinical practice.

## Abstract (sr)

**Uvod:**

Cilj je procena biohemijskog i kliničkog značaja terapije venskim pritiskom u prevenciji venske tromboembolije (VTE) kroz analizu biomarkera koagulacije i fibrinolize, uključujuci fibrinogen (FIB), D-dimer (D-D) i aktivirano parcijalno tromboplastinsko vreme (APTT).

**Metode:**

Randomizovana kontrolisana ispitivanja objavljena između 2013. i 2025. godine sistematski su preuzeta iz baza podataka PubMed, CNKI, VIP i Wanfang. Studije koje su ispunjavale uslove ispitivanja istraživale su terapiju venskim pritiskom i izveštavale o indeksima povezanim sa koagulacijom. Zbirne veličine efekata izračunate su za ključne biohemijske markere (FIB, D-D, APTT, PT, TT) i ishode venske hemodinamike.

**Rezultati:**

Devetnaest kliničkih studija ispunilo je kriterijume za uključivanje. Meta-analiza je pokazala da terapija venskim pritiskom značajno smanjuje nivoe FIB i D-D u plazmi, produžava APTT, protrombinsko vreme (PT) i trombinsko vreme (TT), i poboljšava brzinu protoka venske krvi. Ove promene odražavaju poboljšanu antikoagulantnu aktivnost, pojačanu fibrinolizu i smanjen rizik od tromboze. Važno je napomenuti da je primecena modulacija biohemijskih markera bila u korelaciji sa manjom incidencom duboke venske tromboze donjih ekstremiteta.

**Zaključak:**

Terapija venskim pritiskom povoljno menja biomarkere koagulacije i fibrinolitike, ističuci njihovu dijagnostičku vrednost u pracenju terapijske efikasnosti i trombo-tičnog rizika. Ovi nalazi ističu ključnu ulogu laboratorijskih indeksa u vođenju prevencije i lečenja venske tromboembolije (VTE), podrzavajuci njihovu integraciju u standardizovanu kliničku praksu.

## Introduction

Venous thromboembolism (VTE) is characterized by abnormal coagulation of venous blood within the lumen of deep veins, followed by adhesion to and accumulation along the vessel wall, ultimately obstructing venous outflow. Clinically, this can manifest as lower-limb varicosities or severe pain and poses a substantial threat to both survival and quality of life [1] [2]. The principal manifestations of VTE are lower-extremity deep vein thrombosis (DVT) and pulmonary embolism (PE); In severe cases, these conditions can lead to major adverse clinical events, Including death [3]. The pathophysiology of VTE Is driven primarily by Virchow's triad - sluggish blood flow, endothelial Injury, and hypercoagulability. Surgical procedures and fractures are major précipitants that promote hypercoagulability and related pathological processes. Accordingly, prompt anticoagulation Is central to risk reduction In VTE, and among adjunctive measures, venous pressure-based therapies have demonstrated notable antithrombotic effects [4] [5].

At present, the efficacy and safety of venous pressure therapy In the prevention of VTE remain under In-depth Investigation, and conclusions are not yet consistent. Meta-analysis, by pooling data from multiple studies, can effectively Increase sample size and thereby enhance statistical power. Therefore, this study alms to evaluate the preventive effect of venous pressure therapy on VTE through a meta-analysis, providing an evidence base for Its broader clinical application In VTE prevention.

## Materials and methods

### Literature search

The study design was randomized controlled trials (RCTs). Literature was searched In domestic and International databases Including VIP, CNKI, PubMed, and Wanfang Medical. Search keywords Included: »venous pressure therapy«, »Intermittent pneumatic compression therapy,« »air pressure waves,« »deep venous thromboembolism of the lower extremity«, »prevention,« as well as their Chinese equivalents. The search period covered 2013 to the present. Retrieved studies were carefully read and screened; duplicate content and studies with Inconsistent methods were excluded. Meta-analysis was conducted using RevMan 5.2 based on the final set of high-quality Included studies.

### Inclusion and exclusion criteria

Inclusion criteria: Controlled trials that used Intermittent pneumatic compression or air-pressure wave venous pressure therapy as the Intervention; Studies published from 2013 to the present that followed randomization principles, with no restrictions on nationality, age, sex, or race of participants; Loss to follow-up rate < 20%; Included one or more of the predefined outcome measures; After random allocation, baseline variables other than sample size were comparable between groups.

Exclusion criteria: Case reports, reviews, metaanalyses, conference abstracts, and similar article types; In vitro or animal studies; Missing data for the Investigated outcome Indicators; Studies not approved by relevant Institutions or without participant consent.

### Outcome measures

Incidence of lower-extremity deep venous thromboembolism after Intervention; activated partial thromboplastin time (APTT); plasma fibrinogen (FIB); D-dlmer (DD); prothrombin time (PT); thrombin time (TT); left femoral venous flow velocity; right femoral venous flow velocity; mean venous blood flow velocity In the lower limbs.

### Quality Assessment

The quality of randomized controlled trials was assessed using the modified Jadad scale. This Instrument yields a total score ranging from 1 to 7 points, with scores 3 Indicating low quality and scores 4 Indicating high quality.

### Quality control

A systematic search was conducted based on predefined titles and keywords to ensure comprehensive and accurate literature retrieval. The study team planned to proactively consult recognized experts In the field for professional guidance. When unclear results or missing data were Identified In Included studies, the authors were to be contacted promptly to request supplementation or correction, ensuring completeness and accuracy. Additionally, the relevance and quality of Included studies were rigorously reviewed to confirm the presence of appropriate approval documents, thereby ensuring scientific rigor and compliance.

### Statistical analysis

Data were entered Into RevMan 5.2 for analysis. Dichotomous variables were expressed as risk ratio (RR). Continuous variables were expressed as weighted mean difference (WMD) or standardized mean difference (SMD). All effect sizes were reported with 95% confidence Intervals (Cl). Heterogeneity among studies was assessed using the Chi-square test. When heterogeneity met P<0.1 and I^2^ 50%, a random-effects model was used; otherwise, a fixed-effects model was applied.

## Results

### Literature search results and characteristics

**Figure 1:**
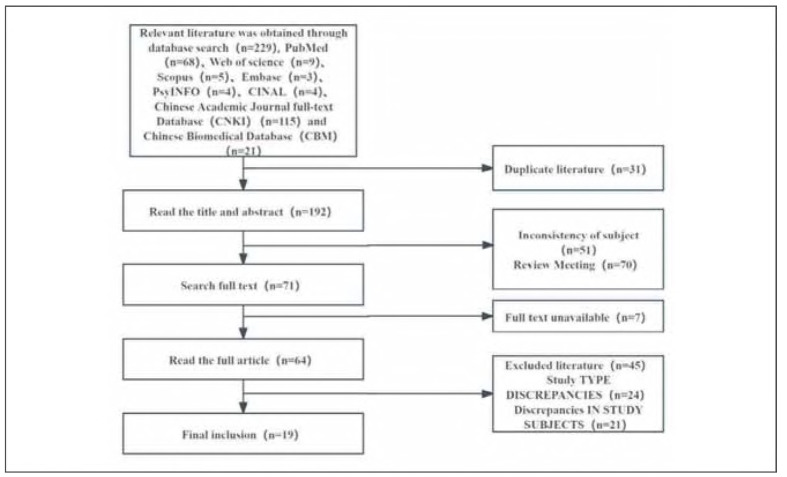
Literature Selection Flowchart.

A comprehensive search of Chinese and English databases - including Wanfang, CNKI, and PubMed - was performed using terms aligned with the study's topic, keywords, and predefined selection criteria. In total, 229 records were identified. After screening according to the inclusion and exclusion criteria, 19 studies met the eligibility requirements, comprising 17 Chinese-language studies and 2 English-language studies. The detailed screening process is presented in Figure 1.

In total, 15 high-quality studies and 4 low-quality studies were included. The characteristics of the included studies and the results of the quality assessment are presented in Table 1 ([6] [7] [8] [9] [10] [11] [12] [13] [14] [15] [16] [17] [18] [19] [20] [21] [22] [23] [24]).

**Table 1:** Comparison of HLA-B27, ESR, and CRP levels between AS patients and healthy controls. Notes: 1 Incidence of lower-extremity deep venous thromboembolism (DVT/VTE) 2 APTT (Activated Partial Thromboplastin Time) 3 FIB (Plasma Fibrinogen) 4 DD (D-dimer) 5 PT (Prothrombin Time) 6 TT (Thrombin Time) 7 Left femoral venous flow velocity 8 Right femoral venous flow velocity 9 Mean venous blood flow velocity of the lower limbs

Name	Year of Publication	Sample Size	Outcomes	Quality Score
Zeng TT	2018	60 /60	12356	4
Chen YJ	2018	24 /24	12356	4
Chen FM	2023	32/32	14	3
Bai XY	2022	100 /100	12345678	7
Shao LH	2019	40 /40	19	2
Cui Y	2021	108/92	1234569	6
Liu ST	2019	93 /93	1234569	6
Sun WL	2019	58 /58	149	3
Li RJ	2019	40 /40	1356	4
Shu YS	2021	41/41	1234578	6
Gui PG	2018	40 /40	12359	5
Wen W	2021	30 /30	1245	4
Ma S	2023	41 /43	12459	5
Gong S	2020	65/65	1234578	6
Chen ZX	2021	200 /200	1234569	6
Ma LY	2018	40 /40	123569	6
Huang YY	2023	41/41	12345678	7
Prell J	2018	41 /53	1	2
Wang JP	2013	60 /60	1234578	4

### Publication bias

**Figure 2:**
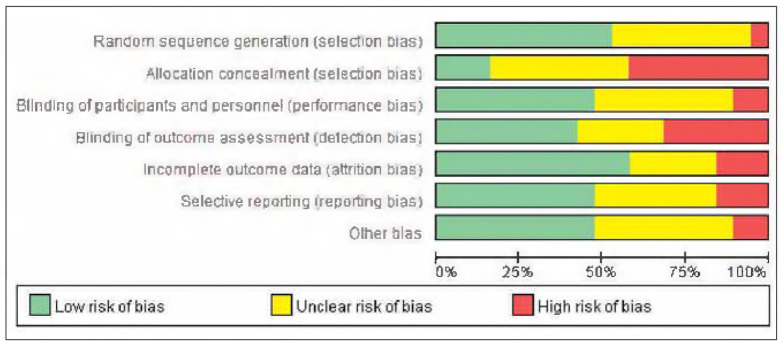
Overall publication bias plot.

**Figure 3:**
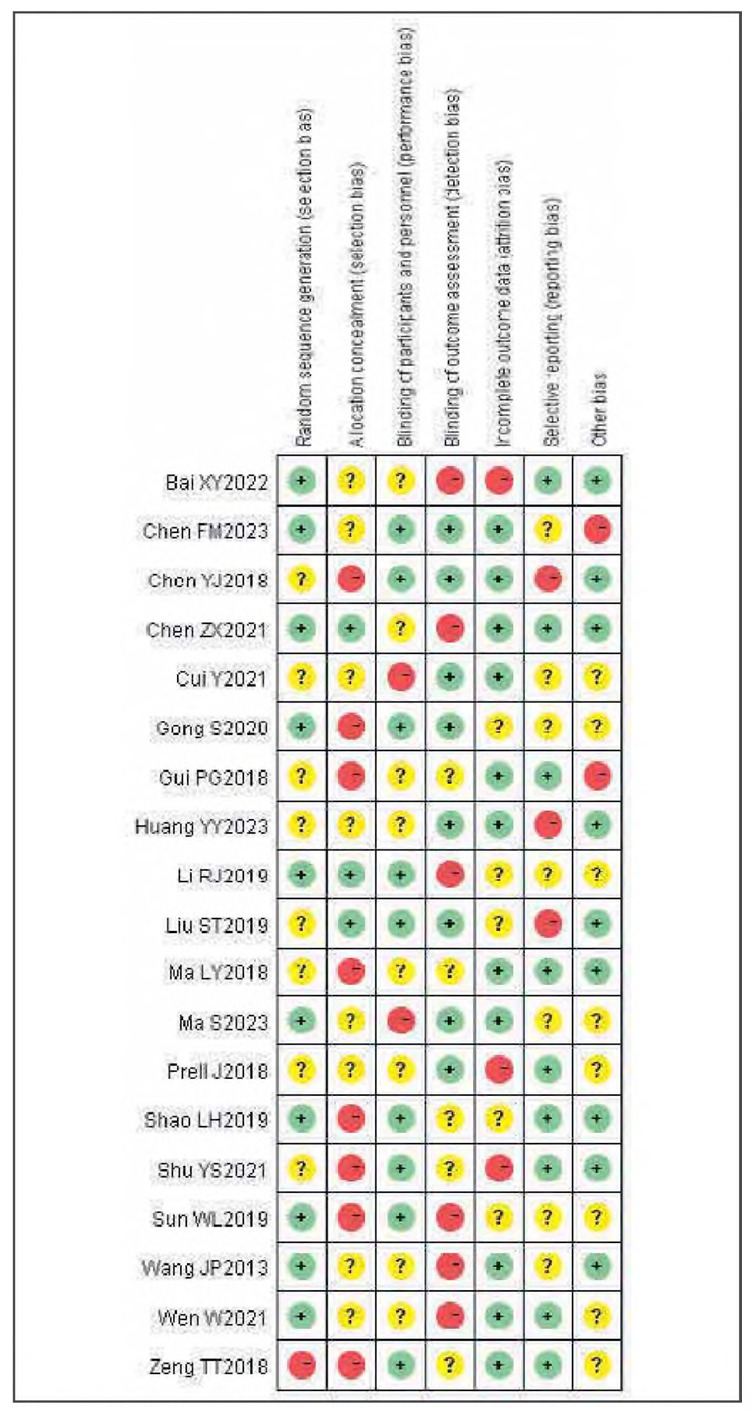
Publication bias plot for individual studies.

There was no apparent publication bias among the 19 included studies. See Figure 2 and Figure 3.

### Incidence of lower limb deep vein thrombosis: meta-analysis

**Figure 4:**
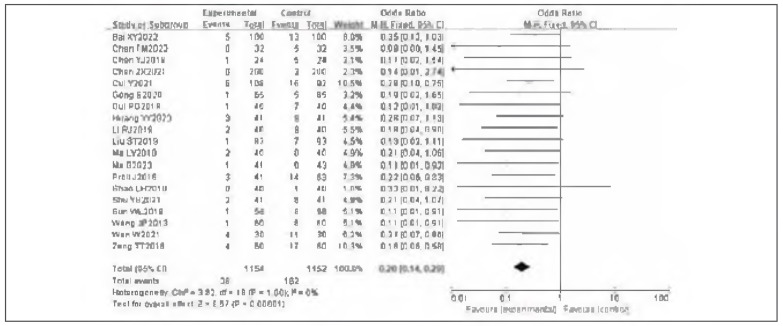
Forest plot of the incidence of lower limb deep venous thrombosis.

**Figure 5:**
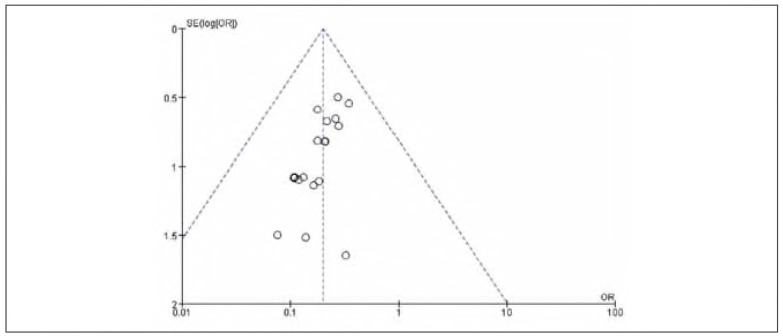
Funnel plot of the incidence of lower limb deep venous thrombosis.

Nineteen studies were included to evaluate the incidence of lower-limb deep vein thrombosis (DVT). Tests for heterogeneity indicated no between-study heterogeneity (I^2^ = 0.0%, P=1.00). Accordingly, a fixed-effect model was applied. The pooled analysis showed that the incidence of lower-limb DVT in the observation group was significantly lower than in the control group, with a statistically significant effect estimate (RR=0.20, 95% Cl:0.14—0.29, P<0.00001). These results indicate that venous pressure therapy significantly reduces the incidence of lower-limb DVT. See Figure 4-Figure 5.

### Meta-analysis of APTT

**Figure 6:**
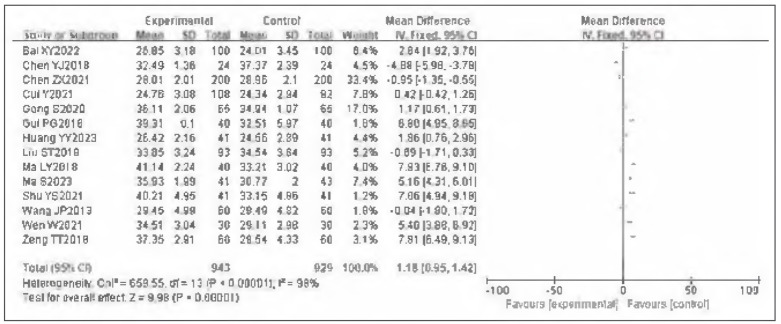
Forest plot of APTT.

**Figure 7:**
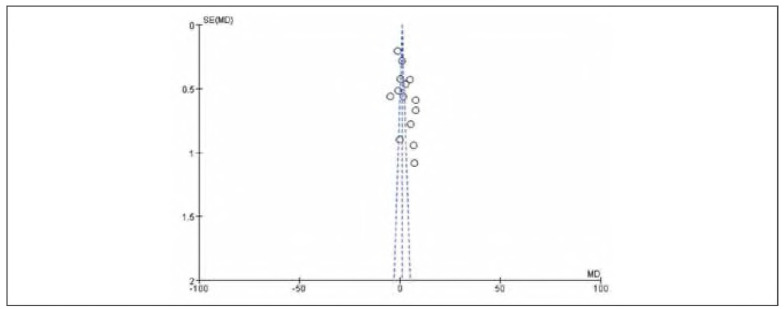
Funnel plot of APTT.

Fourteen studies were included for APTT. Tests for heterogeneity indicated substantial heterogeneity among the studies (I^2^ = 98.0%, P<0.00001). Using a random-effects model, the analysis showed that APTT in the observation group was generally higher than in the control group. The pooled results demonstrated a statistically significant difference (OR=1.18, 95%CI: (0.95, 1.42), P<0.00001). It can therefore be considered that venous pressure therapy increases APTT. See Figure 6-Figure 7.

### Meta-analysis of FIB

**Figure 8:**
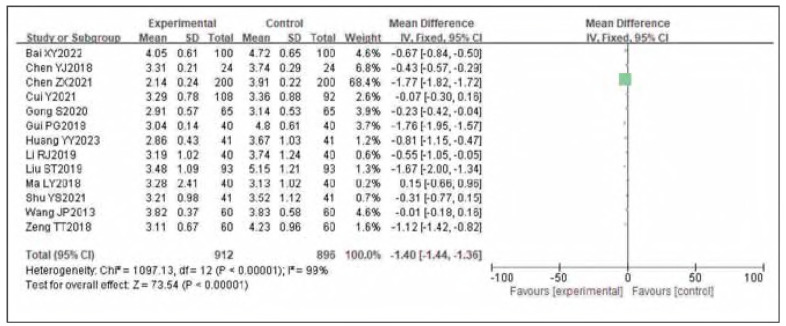
Forest plot of FIB.

**Figure 9:**
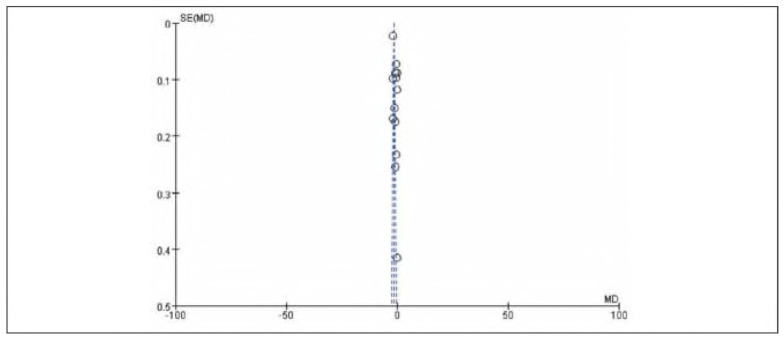
Funnel plot of FIB.

Thirteen studies were included for FIB. Tests for heterogeneity indicated substantial heterogeneity among the studies (I^2^ = 99.0%, P<0.00001). Using a random-effects model, the analysis showed that FIB in the observation group was significantly lower than in the control group. The pooled results demonstrated a statistically significant difference (OR= —1.40, 95%CI: (-1.44, -1.36), P<0.00001). It can therefore be considered that venous pressure therapy reduces FIB. See Figure 8-Figure 9.

### Meta-analysis of DD

**Figure 10:**
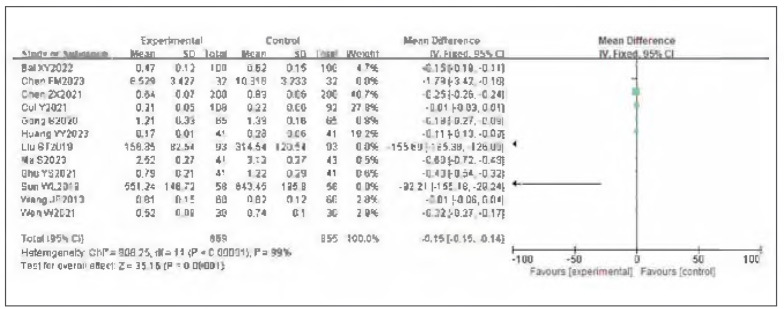
Forest plot of DD.

**Figure 11:**
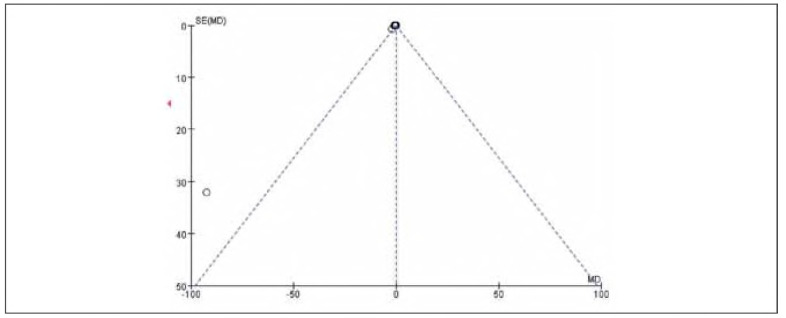
Funnel plot of DD.

Twelve studies were included for DD. Tests for heterogeneity indicated substantial heterogeneity among the studies I^2^ =99.0%, P<0.00001). Using a random-effects model, the analysis showed that DD in the observation group was significantly lower than in the control group. The pooled results demonstrated a statistically significant difference (OR= —0.15, 95%CI: (-0.15, -0.14), P<0.00001). It can therefore be considered that venous pressure therapy reduces DD. See Figure 10-Figure 11.

### Meta-analysis of PT

**Figure 12:**
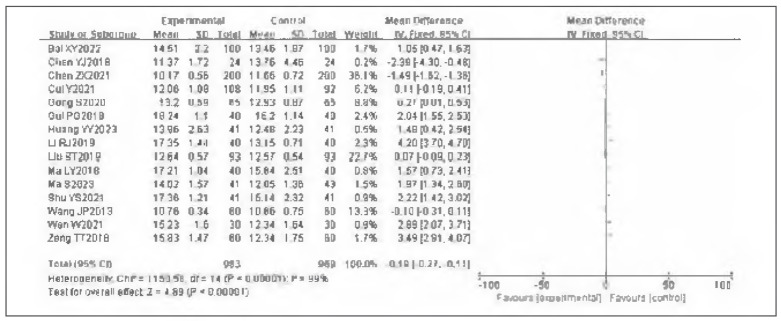
Forest plot of PT.

**Figure 13:**
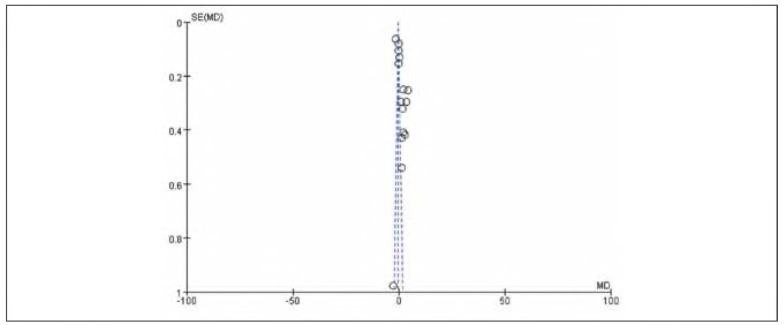
Forest plot of PT.

Fifteen studies were included for PT. Tests for heterogeneity indicated substantial heterogeneity among the studies (l^2^ = 99.0%, P<0.00001). Using a random-effects model, the analysis found that PT in the observation group was significantly higher than in the control group. The pooled results showed a statistically significant difference (OR=—0.19, 95%CI: (—0.27, —0.11), P<0.00001). It can therefore be considered that venous pressure therapy increases PT. See Figure 12-Figure 13.

### Meta-analysis of TT

**Figure 14:**
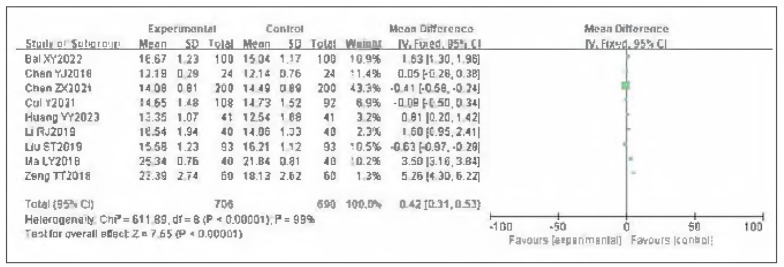
Forest plot of TT.

**Figure 15:**
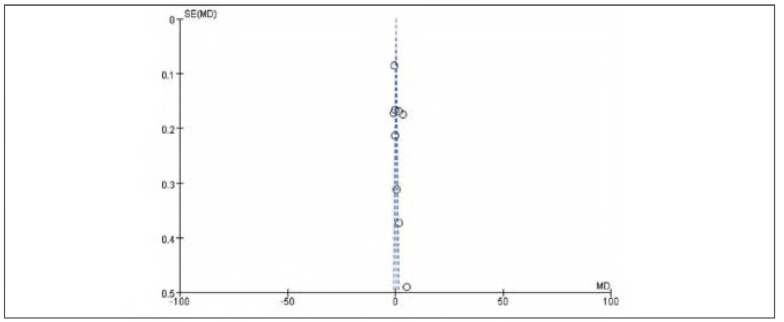
Funnel plot of TT.

Nine studies were included for TT. Tests for heterogeneity indicated substantial heterogeneity among the studies (l^2^ = 99.0%, P<0.00001). Using a random-effects model, the analysis found that TT In the observation group was significantly higher than In the control group. The pooled results showed a statistically significant difference (OR=0.43, 95%CI: (0.31,0.53), P<0.00001). It can therefore be considered that venous pressure therapy Increases TT. See Figure 14-Figure 15.

### Meta-analysis of femoral venous flow velocity

**Figure 16:**
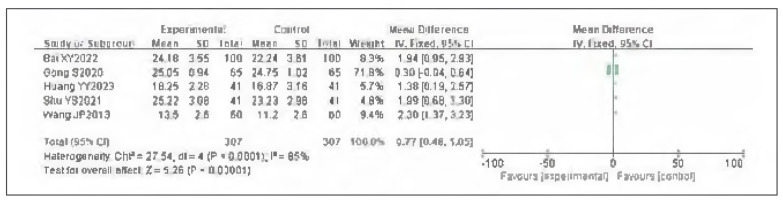
Forest plot of left femoral venous flow velocity.

**Figure 17:**
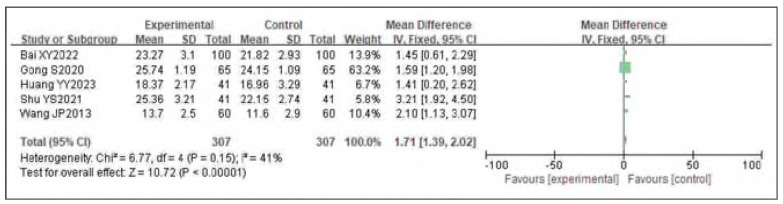
Forest plot of right femoral venous flow velocity.

Five studies were Included for both left and right femoral venous flow velocities. Heterogeneity testing Indicated heterogeneity for one side (I^2^ = 85.0%, P<0.0001) and homogeneity for the other side (I^2^ = 41.0%, P=0.15). Accordingly, random- and fixed-effects models were used, respectively. The analysis found that both left and right femoral venous flow velocities In the observation group were significantly higher than In the control group. The pooled results showed statistically significant differences (OR=0.77, 95%CI: (0.48, 1.05) for one side; OR=1.71, 95%CI: (1.39, 2.02) for the other; P<0.00001). It can therefore be considered that venous pressure therapy Increases both left and right femoral venous flow velocities. See Figure 16-Figure 17.

### Meta-analysis of mean lower-limb venous flow velocity

**Figure 18:**
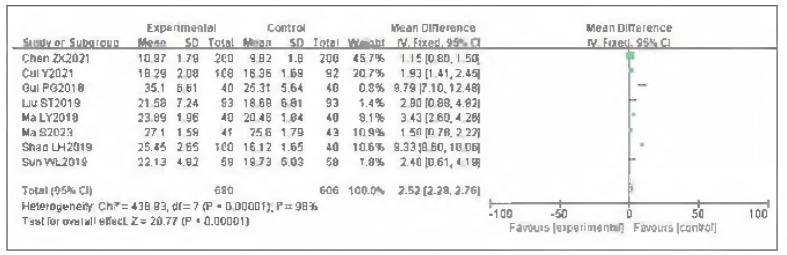
Forest plot of mean lower-limb venous flow velocity.

**Figure 19:**
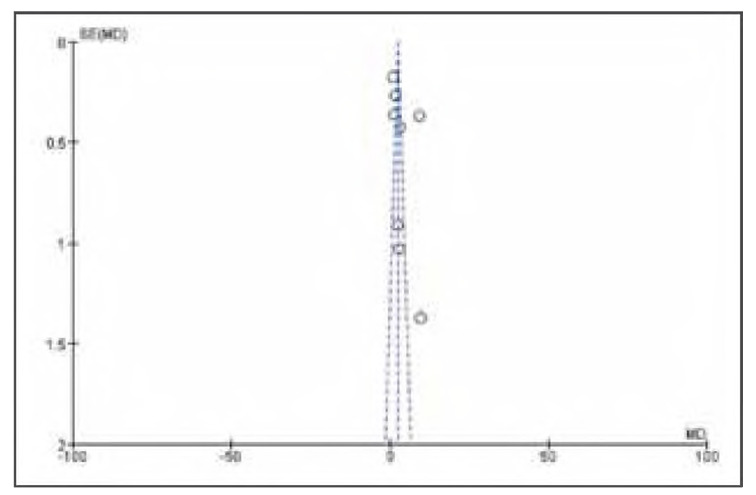
Funnel plot of mean lower-limb venous flow velocity.

Eight studies were Included for mean lower-limb venous flow velocity. Tests for heterogeneity Indicated substantial heterogeneity among the studies (I^2^ = 98.0%, P<0.00001). Using a random-effects model, the analysis showed that the mean lower-limb venous flow velocity In the observation group was significantly higher than In the control group. The pooled results demonstrated a statistically significant difference (OR=2.52, 95%CI: (2.28, 2.76), P<0.00001). It can therefore be considered that venous pressure therapy Increases mean lower-limb venous flow velocity. See Figure 18-Figure 19.

## Discussion

The Incidence of VTE spans from childhood through old age and Increases with advancing age; among Individuals aged 80 years, the Incidence Is approximately 50-fold higher than In younger age groups. Clinical précipitants such as surgery, fractures, and external trauma can disrupt venous blood flow and elevate patient risk [25]. Venography remains the clinical gold standard for diagnosing VTE; however, despite Its high accuracy, It Is Invasive and may cause harm. By contrast, blomarkers provide key adjunctive Information for clinical assessment, and among these, coagulation markers are highly sensitive for predicting and diagnosing thrombosis. Accordingly, evaluating the efficacy of venous pressure therapy using coagulation indices is highly feasible.

In this study, we employed meta-analysis to synthesize findings across different periods, regions, and research teams, thereby constructing a comprehensive and dynamic evidence base. Meta-analysis uses systematic and transparent procedures that enhance the rigor and credibility of the research process. Moreover, because individual study results may vary, meta-analysis offers a structured framework to quantify and interpret between-study heterogeneity [26] [27].

APTT is a sensitive screening indicator commonly used clinically to reflect intrinsic coagulation activity. TT and PT are effective clinical indicators of plasma coagulation factor activity; both can rise significantly when parturients develop coagulation dysfunction or experience massive hemorrhage. D-dimer (D-D) is a degradation product of fibrinolysis; its elevation indicates a hypercoagulable state and enhanced fibrinolysis, showing high sensitivity for early diagnosis of DVT. Fibrinogen (FIB) is a protein involved in both coagulation and hemostasis; in the hypercoagulable state seen in parturients, its level can increase markedly, indicating thrombotic risk [28] [29]. Our results show that after venous pressure therapy, the incidence of VTE is substantially lower, venous blood flow velocities are faster, and coagulation-related indices such as APTT, PT, TT, FIB, and DD improve significantly compared with the control group. These findings further suggest that venous pressure therapy exerts notable anticoagulant effects, alleviates venous congestion, accelerates venous blood flow, and ultimately reduces the risk of VTE, thereby playing a positive preventive role.

The possible mechanisms are as follows: air pressure wave therapy, a common form of venous pressure therapy, can act as a »muscle pump,« propelling venous blood toward the heart and thereby promoting venous circulation. This helps mitigate hypercoagulability and improves fibrinolytic function, jointly producing anticoagulant and VTE-preventive effects. Intermittent pneumatic compression (IPC) is another effective venous pressure therapy that promotes lower-limb venous circulation by cyclically modulating pressure and accelerating the clearance of venous stasis. This generates pulsatile flow and enhances venous return in the distal deep venous systern of the limbs, thereby reducing the aggregation of coagulation factors and their adhesion to the vascular endothelium, fundamentally lowering the risk of thrombus formation and vascular occlusion. Additionally, IPC can inhibit activation of coagulation factors such as FIB while promoting activation of tissue plasminogen activator, thus stimulating the fibrinolytic system, facilitating rapid thrombus clearance, and suppressing thrombogenesis.

This meta-analysis has several limitations. Only three English-language studies were included. We did not perform subgroup analyses based on different timing parameters of venous pressure therapy, leaving room to refine study design for evaluating its preventive effects on lower-limb deep vein thrombosis. Future work should extend the search timeframe for broader literature retrieval, increase the number of included studies, and conduct more in-degth analyses on larger samples. Furthermore, when I^2^ 95%, heterogeneity across studies is extremely high, which may mask biases in some studies or complicate the impact of bias, potentially affecting the consistency of pooled results. Subsequent research should perform subgroup analyses based on baseline characteristics and differences in interventions and apply subgroup analysis and meta-regression to explore sources of between-study heterogeneity.

## Conclusion

In summary, venous pressure therapy exerts significant anticoagulant and fibrinolytic effects, as reflected by improvements in key biochemical markers such as fibrinogen, D-dimer, APTT, PT, and TT. These laboratory indices provide objective evidence of enhanced coagulation balance, improved venous return, and reduced thrombotic risk. By integrating biochemical monitoring with clinical outcomes, our findings underscore the diagnostic value of coagulation markers in evaluating therapeutic efficacy and guiding the standardized application of venous pressure therapy in high-risk populations.

## Dodatak

### Conflict of interest statement

All the authors declare that they have no conflict of interest in this work.
